# Genetic Heterogeneity of Beta Globin Mutations among Asian-Indians and Importance in Genetic Counselling and Diagnosis

**DOI:** 10.4084/MJHID.2013.003

**Published:** 2013-01-02

**Authors:** Ravindra Kumar, Kritanjali Singh, Inusha Panigrahi, Sarita Agarwal

**Affiliations:** 1 Department of Genetics, Sanjay Gandhi Post Graduate Institute of Medical Sciences (SGPGIMS), Lucknow; 2 Genetic-Metabolic unit, Department of Pediatrics, Post Graduate Institute of Medical Education and Research (PGIMER), Chandigarh

## Abstract

There are an estimated 45 million carriers of β-thalassemia trait and about 12,000–15,000 infants with β-thalassemia major are born every year in India. Thalassemia major constitutes a significant burden on the health care system. The burden of thalassemia major can be decreased by premarital screening and prenatal diagnosis. The success of prenatal diagnosis requires proper knowledge of spectrum of β-thalassemia mutations. In present study, β-thalassemia mutations were characterized in 300 thalassemia cases from 2007 to 2010 using ARMS-PCR and DNA sequencing. The five most common mutations accounted 78.9% of the studied chromosomes that includes IVS1-5(G>C), Cod 41-42(-TCTT), Cod8-9(+G), Cod16(−C) and 619bp del. Though IVS1-5(G>C) is most common mutation in all the communities, the percentage prevalence were calculated on sub caste basis and found that IVS1-5(G>C) percentage prevalence varied from 25 to 60 in Aroras & Khatris and Thakur respectively. Interestingly Cod41-42(−TCTT) mutation which is the second commonest among the mutations reported was totally absent in Kayasthas and Muslim community. These findings have implications for providing molecular diagnosis, genetic counseling and prenatal diagnosis to high risk couples of β-thalassemia.

## Introduction

Hemoglobin disorders are genetic diseases consisting mainly of hemoglobinopathies and thalassemia, which account for a great proportion of births affected by a genetic disease.^1^ Thalassemia is a heterogeneous group in which the production of normal hemoglobin is partly or completely suppressed as a result of the defective synthesis of one or more globin chains. This disease has a high frequency in the Mediterranean region, Africa, South-East Asia and the Indian subcontinent.^2^ Recent data indicate that about 7% of the world’s population is carrier of β-thalassemia, and 300,000–500,000 children are born each year with the severe form of the disease i.e. thalassemia major (World Bank 2006, report of a joint WHO-March of Dime meeting 2006). Around 12,000–15,000 infants with β thalassemia major are born every year in India which constitutes about 10% of the total live births in the world.^3^

The only permanent treatment of thalassemia is bone marrow stem cell transplantation but it is possible in only 30–40% of thalassemia patients since it requires the human leukocyte antigen (HLA) matched donor. Practically, the main stay of treatment of β-thalassemia major is regular blood transfusions and concomitant iron chelation therapy which is expensive for developing countries. Thus the disease causes significant morbidity and mortality in affected individuals, making prenatal diagnosis an important option for couples at risk of having β-thalassemia major offspring. Prevention of the disease by genetic counseling and prenatal diagnosis has a pivotal role in thalassemia control in countries like India where government support for thalassemia patients is limited.

The disease is complicated by high mutation rate and presence of genetic modifiers. More than 300 mutations (http://globin.cse.psu.edu/) are known to cause β-thalassemia. Spectrum of mutations varies significantly between different geographical regions and community/ethnic groups.^4^–^10^ The success of prenatal diagnosis requires proper knowledge of spectrum of β-thalassemia mutations, the associated severity and intricacies of interpretation of thalassemia screening results.

## Material and Methods

The present study was undertaken with the objective to determine the frequencies of β-thalassemia mutations and their distribution in various castes and sub castes of Uttar Pradesh, largest state of North India. The study included a total number of 300 thalassemia cases attending Genetics outpatient department (OPD) of a tertiary care center during the period 2007 to 2010. All the patients were clinically evaluated and later referred to genetic hematology laboratory for biochemical and molecular evaluation. Subjects were divided into four groups: thalassemia major, thalassemia intermedia, thalassemia trait, and structural Hb variant groups depending on clinical presentation, transfusion frequencies and the hemoglobin electrophoresis pattern. HPLC was done on Biorad variant HPLC system (Biorad laboratories, Hercules, CA). Criteria to distinguish thalassemia intermedia from major were as follows: (1) age more than 2 years at presentation, (2) no history of regular transfusion, (3) history of acceptable growth even without transfusion and Hb levels of 7–9 g/dl.^11^ A detailed clinical proforma with the information of origin of the family, ethnicity and consanguinity were recorded at the time of clinical evaluation of the patients.

For DNA analysis, 2 ml of blood was drawn and collected in EDTA coated vials from each individual. DNA was extracted from peripheral blood leucocytes by standard phenol chloroform method [Poncz et al 1982]. The common five mutations were characterized by ARMS-PCR method^8^ on PCR system (GeneAmp 9600, Applied Biosystem). Further mutations were detected by DNA sequencing using automated DNA Sequencer (ABI310 Genetic Analyser, Applied Biosystem), as per previously described protocol.^12^

## Results

In the studied cohort, there were 40 cases of thalassemia major, 59 cases of thalassemia intermedia, 146 cases of thalassemia trait and 55 cases of structural Hb variants. Thalassemia intermedia group was further divided into 4 categories i.e. homozygous thalassemia intermedia(12), heterozygous thalassemia intermedia(10), HbE-β thalassemia(23), HbS-β thalassemia(11), and HbD-β thalassemia(3).

The five common mutations accounted for 78.9% of the total chromosomes. Five common mutation found in present study were IVS1-5 (G>C) [47.0%], Cod41-42(−TCTT) [9.3%], Cod8-9(+G) [8.3%], Cod16(−C)[8.3%] and 619bp del[6.0%].

**Figure 1:**
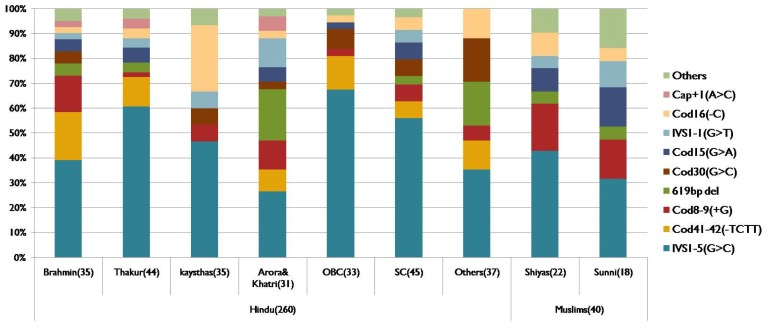
Spectrum of beta thalassemia mutations in different ethnic groups in Asian-Indians

DNA sequencing revealed the less common mutations like: Cod15(G>A) [5.6%], IVS1-1(G>T) and Cod30(G>C) [4.6%] each and Cap+1(A>C) [1.7%]. Other rare mutation comprised of 4.6%. Fig 1 shows the spectrum of beta thalassemia mutations detected in present study.

Mutation spectrum of beta thalassemia was subclassified in various ethnic groups in Hindus like Brahmins, Thakur, Kayasthas, Arora & Khatri, OBC, SC, Others and in Muslims like Shiya and Sunni (Figure 1). It was found that different ethnic groups have different sets and different frequencies of common mutations. Highest heterogeneity was observed in Brahmins and Thakur communities compared to other ethnic groups. The mutation present on first intervening sequence [IVS1-5(G>C)] was found the most common one in all the ethnic groups with a variable frequency from 25% in Arora & Khatri to 60% in Thakur. Interestingly Cod41-42(−TCTT) mutation; which is among the five common mutation in all communities; was totally absent in Kayasthas and Muslim community. 619bp deletion mutation was common in Arora and Khatri groups but it is absent in Kayasthas and OBC groups. The Cod15 (G>A) mutation was found as third most common mutation in Sunni group.

## Discussion

The inherited disorders of hemoglobin synthesis are the most common monogenic disorders worldwide. Thalassemia especially β-thalassemia is a common genetic disorder found in the Indian subcontinent. Since thalassemia is difficult to cure and and management involves major financial inputs, the prevention is a priority. The only cure for affected children is bone marrow transplantation which is management involves major financial inputs, the prevention is a priority. The only cure for affected expensive, risky and available only at selected centers. The only treatment to sustain life in thalassemia major is regular blood transfusion with iron chelation but this requires much commitment on part of the family. The treatment is also hampered by less of blood resources available and lack of motivated voluntary donors. Hence, the most effective approach for developing countries like India is preventing thalassemia. This is possible by targeted carrier detection, genetic counseling and prenatal diagnosis. Thalassemia intermedia is a heterogenous group with most patients being homozygous β-thalassemia with milder β-globin mutations, or with concomitant α-globin gene deletions or XmnI polymorphism. Some patients of thalassemia intermedia are heterozygous β-thalassemia with α-globin gene triplication/quadruplication etc, or rarely dominant β-thalassemia. A co-existence of β-thalassemia heterozygous state with structural hemoglobin variants like HbE, HbS, and HbD also leads to thalassemia intermedia phenotype. HPLC and mutation analysis help in classifying and differentiating these conditions.

Initial studies^13^ have reported the presence of five common β-globin mutations covering approximately 88% of mutations in Indian population residing in west. Later the frequencies of these five common mutations in the Indian subcontinent were determined as 93.6% by Varawalla et al,^14^ 91.8% by Verma et al,^5^ 91.2% by Gupta et al,^15^ 74.5% by Edison et al,^9^ 87.5% by Nadkarni et al.^16^ and 80.0% by Colah et al.^7^

Specifically in northern part of India the frequency of detection of five common mutations was determined as 76.3%, 90.7% and 91.2% by Agarwal et al.^17^–^18^ and Gupta et al.^15^ respectively in the state of Uttar Pradesh (UP). The frequency of detection was 87.2% and 93.1% reported by Garewal et al.^22^–^23^ In our overall analysis in present study, we observed that the five common mutations account for 78.9% of total β-globin gene mutations.

Varawalla et al.^19^ had defined the spectrum of five common β-thalassemia mutations in Indian subcontinents as IVS1-5(G>C), Cod8-9(+G), IVS1-1(G>T), Cod41-42(−TCTT) and 619bp deletion whereas less common mutations were Cod15 (G>A), Cod16 (−C), Cap+1(A>T), Cod30 (G>C), -88(C>T). Later Agarwal et al.^17^–^18^ and Verma et al.^5^ reported the spectrum of less common mutations in North Indians as only Cod15(G>A), Cod16(−C) and Cap+1(A>C) mutations. The variability may be due to variable representation of different ethnic groups in various studies.

Rare mutations which have been described by Varawalla et al.^19^ in Asian Indians were namely −25bp del, Cod5 (−CT), IVS1-1(G>A) and IVSII-837 (G>T). Agarwal et al.^18^ described rare mutation as Cod30 (G>C), Cod5 (−CT), Cod47-48 (+ATCT), IVS1-1 (G>A) present in the population of North Indian origin. Till date 25bp deletion and IVSII-837(G>T) mutation have not been reported from UP state. Gupta et al 2003 reported the less common mutations which included Cod15 (G>A), Cod16 (−C), Cap+1 (A>C) and Cod30 (G>C).

**Figure 2:**
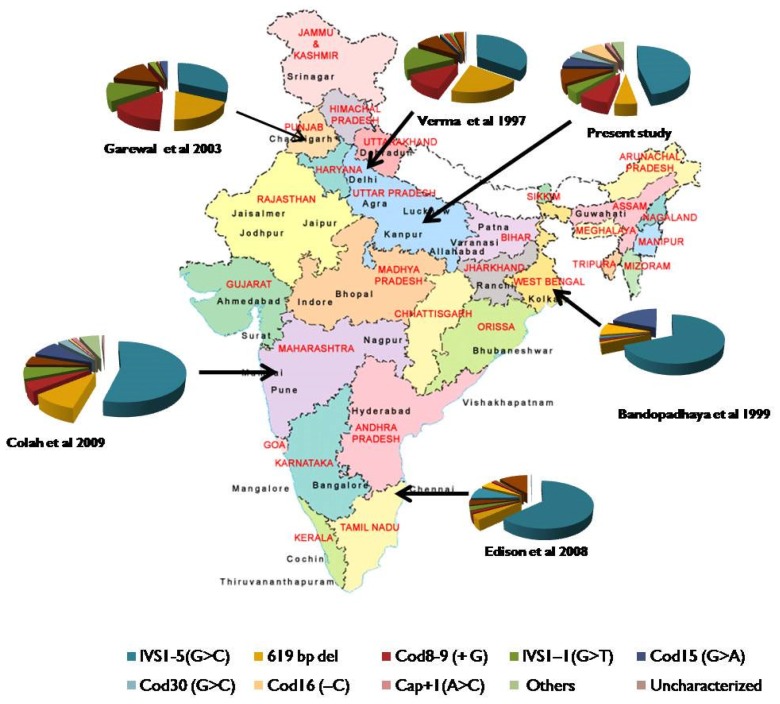
The beta globin gene mutations reported from different regions of India.

Nadkarni et al.^16^ and Colah et al.^20^ reported different spectrum of five common mutations from Central India, and this included IVS1-5 (G>C), 619bp deletion, Cod15 (G>A), IVS1-1(G>T), and Cod8-9(+G). Less common mutation reported by them were Cod41-42(−TCTT), Cod30 (G>C), Cod16 (−C) and Cap+1 (A>C). These differences in the spectrum of mutations in different part of the Indian subcontinent (Figure 2) represent different ethnicities, and different endogamy patterns prevailing in the country.

All the previous studies reported Cod16 (−C) as less common or rare mutation but in present study frequency of Cod16 (−C) is very high in population of Uttar Pradesh state. Interestingly the frequency of IVS1-1 (G>T) mutation is found remarkably low in the present study and found position in less common mutation, though it was one of five common mutations especially from Punjab state.

Cod30 (G>C) earlier considered as a rare mutation was found to be a less common mutation in present study. Conversely the promoter mutation -88 (C>T) found to be rare, milder mutation in UP population, though it is frequent in Jat-Sikhs from Punjab.^21^ It is interesting to note that Cod47-48 (+ATCT) mutation reported earlier were not observed in the present analysis. Thus the spectrum of mutations can vary depending on whether we are studying only thalassemia major patients, or we are including other cases of homozygous beta-thalassemia presenting as thalassemia intermedia.

Though IVS1-5 (G>C) is most common mutation in Indians, its percentage prevalence varies from 25% in Arora & Khatri to 60% in Thakur community. Interestingly Cod41-42(−TCTT) mutation and 619bp del mutation was totally absent in Kayasthas. The Cod15 (G>A) mutation was common in Sunni community where it was found as third most common mutation.

Thus, the present study provides information on the distribution of the β-thalassemia mutations in β-globin gene in the multiethnic population of Uttar Pradesh state. The application of the knowledge of ethnic origin and mutation pattern would help to reduce the screening cost and also facilitate early and better genetic counseling of families and high risk couples.
